# PTEN status in advanced colorectal cancer treated with cetuximab

**DOI:** 10.1038/sj.bjc.6605471

**Published:** 2009-12-01

**Authors:** F V Negri, C Bozzetti, C A Lagrasta, P Crafa, M P Bonasoni, R Camisa, G Pedrazzi, A Ardizzoni

**Affiliations:** 1 Medical Oncology Unit, University Hospital, Parma, Italy; 2 Pathology Department, University Hospital, Parma, Italy; 3 Pathology Department, Arcispedale ‘S. Maria Nuova’, Reggio Emilia, Italy; 4 Department of Public Health, University of Parma, Parma, Italy

**Keywords:** PTEN, colorectal cancer, cetuximab

## Abstract

**Background:**

Loss of phosphatase and tensin homologue deleted in chromosome 10 (PTEN) function in advanced colorectal cancer (CRC) may represent one of the resistance mechanisms to cetuximab by interfering with the epidermal growth factor receptor signal transduction pathway.

**Methods:**

PTEN expression tested by indirect immunofluorescence was evaluated both on primary (*n*=43) and on metastatic (*n*=24) sites in CRC patients treated with cetuximab.

**Results:**

The loss of PTEN expression tested on metastatic sites was negatively associated with response (100% progressive disease (PD) in PTEN-negative cases *vs* 30% PD in PTEN-positive cases; *P*<0.05), PFS (0.8 *vs* 8.2 months; *P*<0.001) and OS (2.9 *vs* 14.2 months; *P*<0.001).

**Conclusion:**

A potential role of PTEN in the anti-tumour activity of cetuximab could be hypothesised.

Among patients with advanced colorectal cancer (CRC), a small subgroup seems to benefit from the epidermal growth factor receptor (EGFR) inhibitor, cetuximab (Cunningham *et al*, 2004; Saltz *et al*, 2004). An optimal response to EGFR inhibitors requires the EGFR-activated intracellular signal transduction pathway to be intact (Ciardiello and Tortora, 2008). Epidermal growth factor receptor-dependent cancer cells may escape from cetuximab inhibition by using alternative pathways (Viloria-Petit *et al*, 2001) or through a continuous activation of downstream intracellular signalling (Bianco *et al*, 2003).

Phosphatase and tensin homologue deleted in chromosome 10 (*PTEN*) is an important tumour-suppressor gene that negatively regulates Akt activities (Stambolic *et al*, 1998). Loss of PTEN function has been reported in CRC (Thomas and Grandis, 2004) and may represent one of the resistance mechanisms interfering with the response to EGFR antagonists by dissociating EGFR inhibition from the downstream phosphatdylinositol 3-kinase/Akt pathway (Baselga, 2001).

## Materials and methods

Our experience refers to 50 mCRC patients submitted both to the evaluation of PTEN and pAKT expression by indirect immunofluorescence (IFI) and to *PTEN* and *EGFR* gene copy number assessments by fluorescence *in situ* hybridisation (FISH). Phosphatase and tensin homologue deleted in chromosome 10 and pAKT IFI assessments were performed on 4-*μ*m-thick tissue sections of paraffin-embedded specimens by using a PTEN rabbit monoclonal antibody (Millipore, Billerica, MA, USA) and a pAKT (Ser 473) rabbit monoclonal antibody (Cell Signaling Technology, Beverly, MA, USA), respectively, followed by FITC-conjugated specific secondary antibody (Sigma-Aldrich Corp., St Louis, MO, USA). Indirect immunofluorescence-positive tumour cells were recognised by bisbenzimide (Hoechst 33258) (Sigma-Aldrich Corp.) and images were obtained by fluorescent microscopy (Olympus BX41, Olympus, Inc., Melville, NY, USA). Fluorescence intensity was scored as absent, weak, moderate and strong. For both PTEN and pAKT, the percentage of cells expressing antigens was determined by evaluating the number of positive cells in a field with reference to the total number of cells in that field. Samples with an absent or weak expression in <10% of cells were considered as negative.

Phosphatase and tensin homologue deleted in chromosome 10 copy number status assessment was performed on 4-*μ*m paraffin-embedded sections using a hybridisation solution containing both a rhodamine-conjugated probe that is specific for the *PTEN* locus on chromosome 10q23.21 and a FITC-conjugated control probe specific for 10p11.1–q11.1 (LSI *PTEN*/CEP10 – Vysis Inc., Downers Grove, IL, USA). Nuclei were counterstained with DAPI for viewing on an Olympus MX60 fluorescence microscope with a 100-W mercury lamp. Separate narrow band pass filters were used for the detection of Spectrum Orange, Spectrum Green and DAPI. Hemizygous deletion of *PTEN* was defined as >20% of tumour nuclei containing one *PTEN* locus signal and by the presence of CEP10 signals. Homozygous deletion of *PTEN* was exhibited by the simultaneous lack of both *PTEN* locus signals and by the presence of control signals in >30% of cells (Yoshimoto *et al*, 2007).

Epidermal growth factor receptor FISH was performed using the LSI *EGFR* Spectrum Orange/CEP 7 Spectrum Green probe set (Vysis), and counterstaining and viewing were performed as described above for the *PTEN* gene. An increased *EGFR* gene copy number was defined as the presence of three or more signals per nucleus (Moroni *et al*, 2005).

## Results

Of the patients, 80% (40 out of 50) received ⩾3 lines of chemotherapy. A total of 36 patients were treated with cetuximab and irinotecan and 14 with cetuximab and oxaliplatin. Patients who obtained a partial response (PR) or a stable disease (SD) were defined as responders. In all, 12 patients (24%) experienced PR, 14 (28%) experienced SD and 24 (48%) experienced a progressive disease (PD). At a median follow-up of 23 months, 49 patients (98%) progressed and 40 (80%) died. In the whole group, median PFS and OS were 4.0 and 9.3 months, respectively.

**Figure 1:**
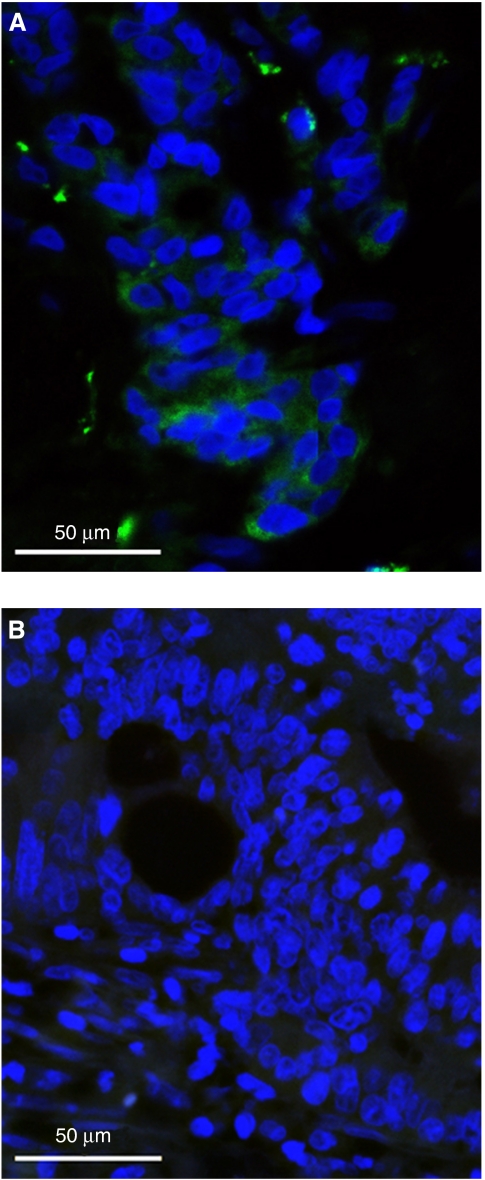
PTEN protein expression by immunofluorescence in CRC cells. (**A**) Positive (green) cytoplasmic staining. (**B**) Negative staining. Scale bar=50 *μ*m.

Phosphatase and tensin homologue deleted in chromosome 10-positive staining was mainly localised to the cytoplasm of CRC cells. An example of a PTEN-positive CRC is shown in Figure 1a and a PTEN-negative CRC in Figure 1b. In all, 5 out of 43 (12%) of the evaluable primary tumours, and 4 out of 24 (17%) of the metastases were PTEN IFI negative. The loss of PTEN expression tested on metastatic sites was negatively associated with response (100% PD in PTEN-negative cases *vs* 30% PD in PTEN-positive cases; *P*<0.05) (Table 1), PFS (0.8 *vs* 8.2 months; *P*<0.001) and OS (2.9 *vs* 14.2 months; *P*<0.001). Of the paired primary tumours and matched metastases, 25% (5 out of 20) were discordant for PTEN expression. Hemozygous deletion of *PTEN* was present in 33% (15 out of 45) of primary tumours and all of these had an absent or weak protein expression (*P*<0.005). The *PTEN* FISH assessed on primary tumour was neither predictive nor prognostic. No correlation was found between *PTEN* FISH and PTEN expression on metastatic sites probably because of the low number of cases (*n*=17). pAKT was not predictive of response, PFS and OS, neither was EGFR.

## Discussion

According to the data obtained by Loupakis *et al* (2009), who evaluated PTEN expression by IHC, we found a significant correlation between PTEN tested by IFI on metastatic sites and response and PFS. Moreover, in our data, PTEN also predicted OS. However, given the small number of cases, our results may deserve further validation in a wider cohort.

In our series, 25% (5 out of 20) of cases showed a PTEN primary tumour *vs* metastasis discordance. As recently reported by Molinari *et al* (2009), our observation confirms that primary CRC and paired metastasis may exhibit a difference with respect to the EGFR pathway; therefore, the analysis of metastatic sites should be considered, given that PTEN assessed on primary tumour might incompletely predict the response.

Epidermal growth factor receptor protein expression is no longer considered as selection criteria for cetuximab sensitivity (Cunningham *et al*, 2004; Saltz *et al*, 2004; Chung *et al*, 2005), and the mechanism of cetuximab's anti-tumour activity remains a fundamental question to be clearly addressed. A possible functional interaction of PTEN activity with EGFR tyrosine kinase signalling and a potential role for PTEN in the anti-tumour activity of cetuximab could be hypothesised.

## Figures and Tables

**Table 1:** PTEN IFI on primary tumours and metastases and response to treatment

	**Primary tumours (*n*=43)**			**Metastases (*n*=24)**		
	**Responders *n* (%)**	**Non-responders *n* (%)**	***P*-value**	**Responders *n* (%)**	**Non-responders *n* (%)**	***P*-value**
PTEN negative	1 (20)	4 (80)		0 (0)	4 (100)	
PTEN positive	21 (55)	17 (45)	0.185	14 (70)	6 (30)	0.02

Abbreviations: PTEN=phosphatase and tensin homologue deleted in chromosome 10; IFI=indirect immunofluorescence.
